# Analysis of the Relationship Between Early Estrogen Response and Cryptorchidism

**DOI:** 10.2174/0118715303419738251111055407

**Published:** 2026-01-15

**Authors:** Dashuai Miao, Yifang Sun, Xiang Yu, Suyin Feng, Long Zhu, Xiao Tong, Wenliang Ge, Hua Li

**Affiliations:** 1 Department of Pediatrics, Donghai County People's Hospital (Affiliated Kangda College of Nanjing Medical University), Donghai, 223000, China;; 2 Jiangnan University Smart Healthcare Joint Laboratory, Donghai County People's Hospital (Affiliated Kangda College of Nanjing Medical University), Donghai, 223000, China;; 3 Department of Neurosurgery, Donghai County People's Hospital (Affiliated Kangda College of Nanjing Medical University), Donghai, 223000, China;; 4 Department of Pediatrics, Jiangnan University Affiliated Hospital, Wuxi, 214122, China;; 5 Department of Pediatric Surgery, Affiliated Hospital of Nantong University, Nantong, China

**Keywords:** Early estrogen response, cryptorchidism, machine learning, biomarker, transcription factor, enrichment analysis

## Abstract

**Introduction:**

Cryptorchidism, a common congenital malformation in male newborns due to abnormal testicular descent, is closely linked to male infertility and germ cell tumors. The study suggested that estrogen response contributes to its occurrence.

**Methods:**

Data were collected from Gene Expression Omnibus (GEO). Enrichment scores were calculated by single-sample gene set enrichment analysis (GSEA), while pathway analysis was performed using GSEA. Key modules were identified through weighted gene co-expression network analysis (WGCNA), followed by the screening of differentially expressed genes (DEGs) using the limma package. The intersection genes were further screened by LASSO and Support Vector Machine-Recursive Feature Elimination (SVM-RFE). Potential transcription factors (TFs) were predicted using hTFtarget, and related microRNAs were analyzed using the Encori database. The TF regulatory network was visualized using Cytoscape 3.8.0.

**Results:**

Patients with cryptorchidism exhibited significantly higher early estrogen response scores compared to controls, with a notable enrichment of the early estrogen response pathway. This study identified 2 biomarkers, *PRR15* and *SRPX*, which were both regulated by the TF *SPI1*. *PRR15* gene expression was significantly related to early estrogen response. Additionally, these two biomarkers were primarily involved in cell division pathways; in particular, *PRR15* was closely linked to progesterone-mediated oocyte maturation.

**Discussion:**

*PRR15* and *SRPX*, which SPI1 potentially regulated, were identified as genes associated with early estrogen response in cryptorchidism and were enriched in estrogen-related and cell division pathways.

**Conclusion:**

This study provided translational insights, enhancing the current understanding of testicular pathogenesis and contributing to the diagnosis and treatment of cryptorchidism.

## 1 INTRODUCTION

Cryptorchidism is a developmental disorder in which one or both testes fail to descend into the ipsilateral scrotum during fetal development due to abnormal testicular positioning or impaired descent mechanisms. This occurs due to disrupted migration along the inguinal canal via the processus vaginalis of the peritoneum [1]. Cryptorchidism is linked to significant long-term health issues, including infertility and a higher risk of testicular cancer [2, 3]. The process of testicular descent is a highly complex phenomenon, primarily occurring during the third trimester of pregnancy and involving intricate hormonal and mechanical interactions [3, 4]. Though cryptorchidism is known to be influenced by both genetic and environmental factors, its precise etiology remains incompletely understood. Therefore, further investigation into the pathogenesis, early diagnostic methods, and effective treatment for cryptorchidism is critically needed.

Genetic factors play a pivotal role in the development of cryptorchidism, with multiple genes and signaling pathways intricately involved in the testicular descent process [1]. Emerging evidence highlights the importance of coiled-coil domain-containing (CCDC) family proteins in reproductive biology. Notably, CCDC149 has been shown to regulate muscle-related genes, potentially affecting bone conduction development and impairing testicular descent [3]. Hormonal factors secreted by the fetal testis, including insulin-like growth factor 3 (*INSL3*) and androgens, are vital for the normal descent of the testis [5, 6]. Mouse model studies have elucidated the crucial role of *INSL3* and its receptor, relaxin family peptide receptor 2 (*RXFP2*), in mediating transabdominal testicular migration [5].

Additionally, estrogen can downregulate the expression of *INSL3*, potentially resulting in testicular hypoplasia [7]. Studies have shown that the male offspring of women who took diethylstilbestrol during pregnancy to prevent miscarriage have a notably higher risk of cryptorchidism [8]. Cryptorchidism is associated with a specific haplotype of the estrogen receptor α gene [9]. In addition, some studies proposed that the inhibitory effect of estrogen on testicular descent and testicular steroidogenesis is mediated through estrogen receptor β [10]. Despite these findings, a significant knowledge gap remains regarding the systematic analysis of estrogen-gene interactions in cryptorchidism. This underscores the urgent need to identify and validate biomarkers related to estrogen response to study the occurrence of cryptorchidism [11, 12].

This study innovatively integrated WGCNA with machine learning algorithms to systematically identify potential biomarkers associated with early estrogen response in cryptorchidism. We first selected candidate genes through a combination of WGCNA and differential expression analysis, and then employed machine learning methods for cross-validation to determine key genes. Finally, functional enrichment and regulatory network analysis were employed to explore the potential impact of key biomarkers on early estrogen response. Together, this study provides a framework for understanding cryptorchidism, contributing to the diagnosis and treatment of this condition.

## 2 MATERIALS AND METHODS

### 2.1 Data Acquisition

This study was a retrospective analysis of publicly available data. The flowchart of the study is shown in Fig. (**S1**). The cryptorchidism datasets GSE16191 and GSE25518 were downloaded from the GEO database (https://www.ncbi.nlm.nih.gov/geo/). Specifically, GSE16191 contained 16 testicular samples from patients with cryptorchidism and 4 contralateral control samples from the same patients. GSE25518 included 19 normal testicular samples and 4 contralateral control samples from patients with cryptorchidism. Information about the patient in both datasets can be found in Table **S1**. The inclusion criteria for this study were cryptorchidism microarray sequencing samples with consistent sampling sites and sequencing platforms; those that did not meet these conditions were excluded. Subsequently, the batch effect was removed using the COMBAT method in the sva package [13], and the two datasets were combined into a single dataset. Early estrogen response-related genes were obtained from the Msigdb database (https://www.gsea-msigdb.org/gsea/msigdb/) using the HALLMARK_ESTROGEN_RESPONSE_EARLY dataset, yielding 200 early estrogen response-correlated genes.

### 2.2 Enrichment Analysis

The enrichment scores for a background gene set of 200 early estrogen response-related and inflammation-associated genes in each sample were computed using single-sample GSEA (ssGSEA) [14]. GSEA_4.2.2 [15] was employed to conduct GSEA on the database set for both cryptorchidism and control samples, with the HALLMARK_ESTROGEN_RESPONSE_EARLY gene set as the background. For the GSEA targeting biomarkers, the background gene sets c2.cp.kegg and c5.go were selected to perform KEGG and GO gene set enrichment analyses, respectively.

### 2.3 Weighted Gene co-expression Network Analysis (WGCNA)

WGCNA [16, 17] was utilized to systematically identify gene modules associated with early estrogen response in cryptorchidism, with a soft thresholding power of 0.85, a minimum of 200 genes per module, and a merge threshold for modules of 0.2 The threshold was determined based on a scale-free topology fit (R^2^ > 0.85), as recommended for gene co-expression networks. Ultimately, modules related to both early estrogen response and cryptorchidism were identified as key modules. The module membership (MM) and gene significance (GS) values were calculated for the genes, with those with |GS| ≥ 0.4 and |MM| ≥ 0.8 being considered as hub genes.

### 2.4 Analysis of DEGs

Differential expression analysis between the cryptorchidism group and the control group was performed using the limma package [18, 19]. Differentially expressed genes (DEGs) were identified using the thresholds |log2FC| ≥ 1 and *p* < 0.05.

### 2.5 Machine Learning for Biomarker Selection

Common genes between the hub genes and DEGs were taken as the candidate genes for further screening by machine learning algorithms. The glmnet package in R [20] was used to perform LASSO regression analysis, and the model with the smallest error was selected as the LASSO analysis result. The e1071 package in R [21] was employed to conduct SVM-RFE analysis on the candidate genes, and RFE selected the model with the minimum error. The genes identified by the two models were intersected to obtain the biomarkers for cryptorchidism.

### 2.6 Construction of TF Regulatory Network for Biomarkers

The potential TFs for the biomarkers were predicted employing the hTFtarget database (https://guolab.wchscu.cn/hTFtarget/#!/). The microRNAs (miRNAs) targeted by the biomarkers were predicted using the Encori database (https://rnasysu.com/encori/), retaining only those verified by at least two experimental validations. The aforementioned predicted results were imported into Cytoscape 3.8.0 [22] to construct a TF regulatory network.

### 2.7 Statistical Analysis

All statistical analyses were conducted using the R language (version 3.6.0), and *p* < 0.05 was considered statistically significant. Sangerbox (http://sangerbox.com/) [23] offered analytical assistance.

## 3 RESULTS

### 3.1 Data Integration and Batch Effect Removal

**Fig. (1):**
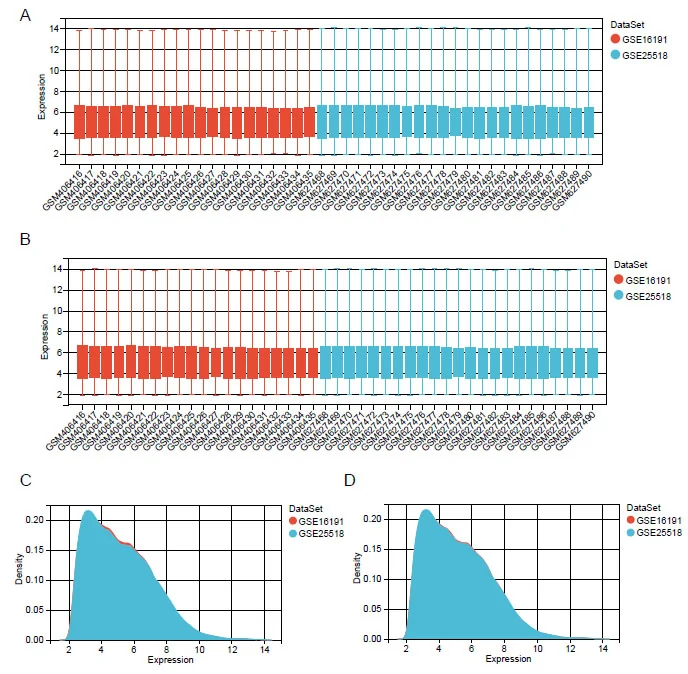
Boxplots and density plots of sample expressions before and after the batch effect removal. (**A**) Boxplot of average sample expression levels before batch effect removal; (**B**) Boxplot of average sample expression levels after batch effect removal. (**C**) Density plot of sample expression levels before batch effect removal. (**D**) Density plot of sample expression levels after batch effect removal.

The boxplots of average sample expression levels before and after batch effect removal (Figs. **1A** and **B**) and the density plots of expression levels (Figs. **1C** and **D**) are shown. Since the datasets were sequenced using the same microarray platform, the batch effect was minimal.

### 3.2 Early Estrogen Response Scores and Enriched Pathways in Cryptorchidism

**Fig. (2):**
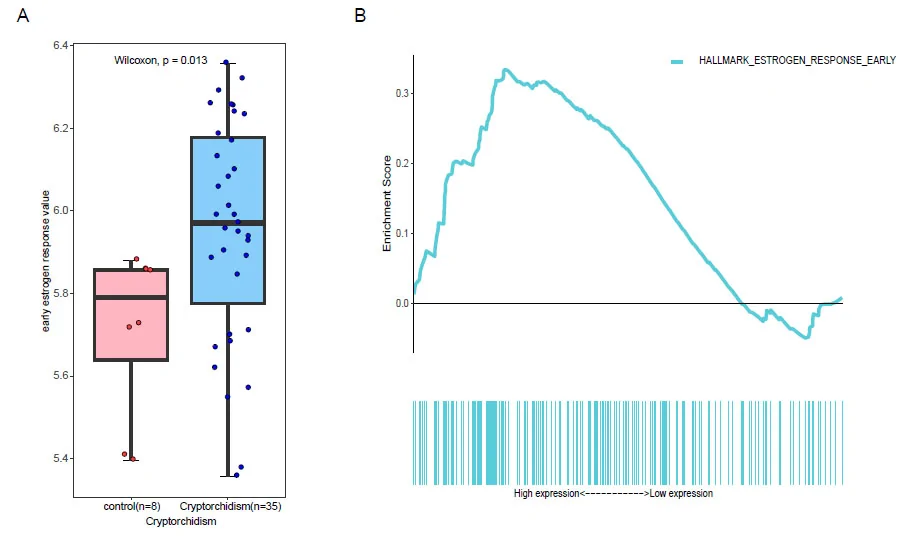
Early estrogen response scores and GSEA enrichment results. (**A**) Boxplot of early estrogen response scores between cryptorchidism and control groups. (**B**) GSEA enrichment results for early estrogen response-related pathways. The top part of the figure displays the calculation process of the enrichment score (ES) value. As we move from left to right, an ES value is calculated for each gene, and these values are connected to form a line. The peaks and troughs formed represent the ES values of the gene set on the phenotype. A positive ES indicates that a functional gene set is enriched at the front of the ranked list, while a negative ES indicates that it is enriched at the back. The bottom part represents the distribution of gene expression levels within the pathway.

Using a set of 200 genes associated with early estrogen response as the background gene set, we calculated early estrogen response scores for each sample. The results showed notably higher early estrogen response scores in cryptorchidism samples than in control samples (Fig. **2A**, *p* < 0.05). GESA results indicated significant enrichment of early estrogen response pathways in cryptorchidism patients (Fig. **2B**).

### 3.3 WGCNA Screened the Hub Genes

**Fig. (3):**
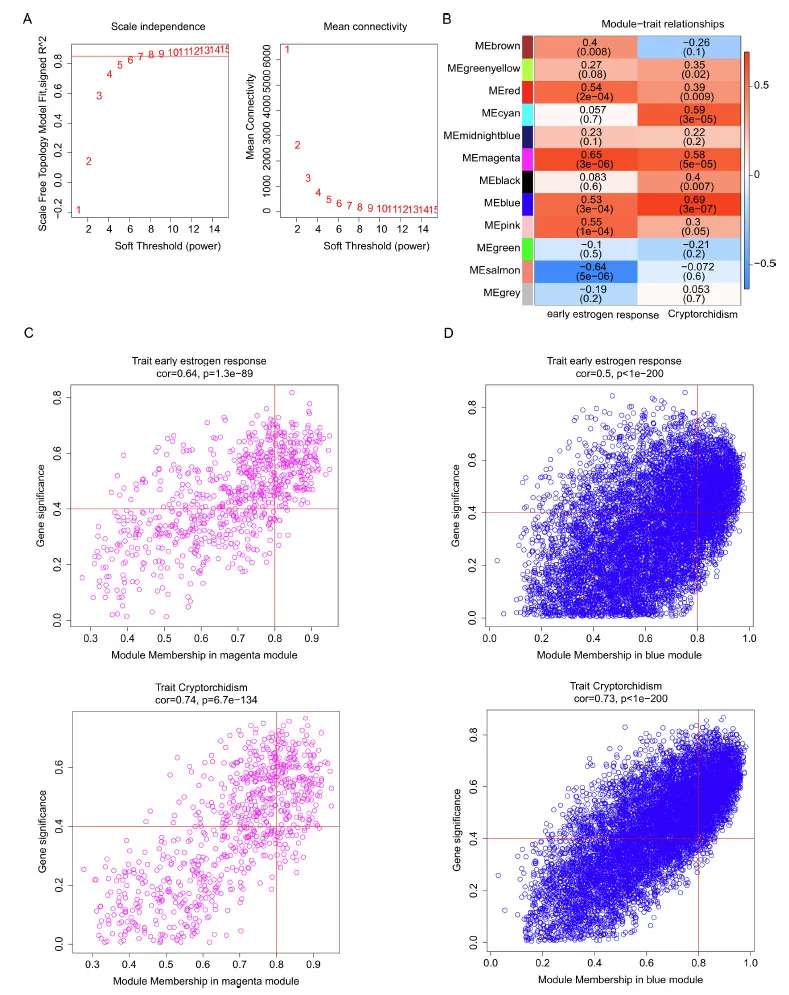
WGCNA screened the hub genes. (**A**) Soft-threshold screening plot. The minimum value of 8, with an R² higher than 0.85, was screened out as the soft threshold for constructing the topological network. (**B**) Module-trait correlation plot. In the plot, the abscissa represents the traits, and the ordinate represents the modules. The numbers in the squares are the correlation coefficients, and the numbers in parentheses are the significance P-values. Blue represents negative correlation, red represents positive correlation. (**C**) GS-MM plot of the magenta module. The abscissa is MM, that is, the correlation of each gene with the characteristic gene, and the ordinate is GS, that is, the correlation of the genes within the module with the trait. (**D**) GS-MM plot of the blue module.

Through WGCNA, we determined an optimal soft threshold power of 8 for constructing the co-expression network (Fig. **3A**). We obtained 12 modules with at least 200 genes after calculating module correlations. Correlation analysis between the modules and traits revealed a positive relationship (greater than 0.5) between the MEmagenta and MEblue modules and both early estrogen response and cryptorchidism (*p* < 0.05, Fig. **3B**). Subsequently, under the criteria of GS ≥ |0.4| and |MM| ≥ 0.8, 1,561 hub genes within these 2 modules were identified (Figs. **3C** and **D**).

### 3.4 Acquisition of DEGs and Candidate Genes

**Fig. (4):**
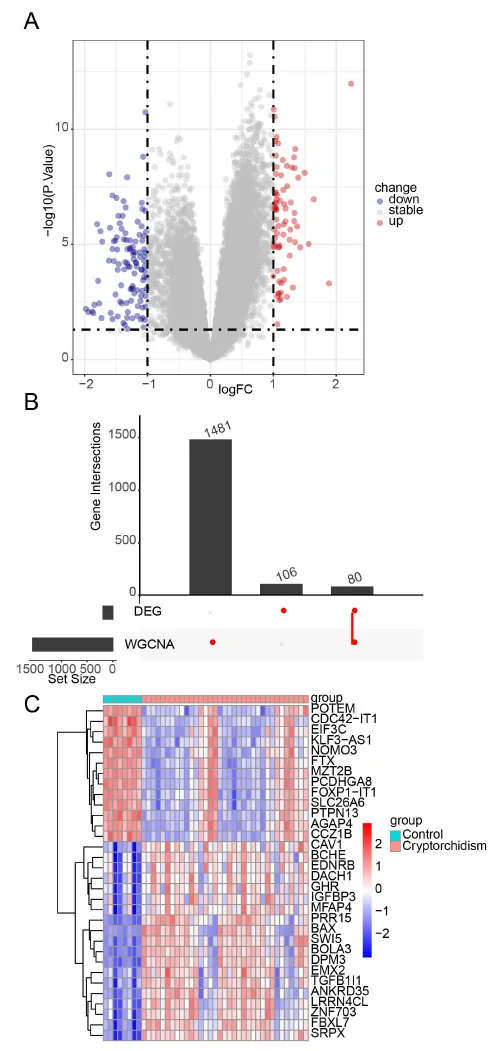
Analysis of DEGs and candidate genes. (**A**) Volcano plot of gene differential expression. The abscissa represents the log2FC of the fold change of differential expression, and the ordinate represents -log10(P.value). Each point in the plot represents a gene, with blue representing down-regulated DEGs and red representing up-regulated DEGs. (**B**) Upset plot of the intersection of WGCNA and DEGs. The upper bar chart shows the number of genes in each intersection, and the left bar chart shows the number of genes in each subset. (**C**) Heatmap of differential expression of candidate genes, with red representing high expression and blue representing low expression.

A total of 186 DEGs (111 downregulated genes and 75 upregulated genes) were obtained through differential analysis (Fig. **4A**). By intersecting the hub genes identified through WGCNA and the DEGs, 80 DEGs related to early estrogen response were acquired (Fig. **4B**). After deleting novel genes and multigene families, 32 candidate genes were identified, and their expression heatmap was generated (Fig. **4C**).

### 3.5 Screening cryptorchidism biomarkers through machine learning methods

**Fig. (5):**
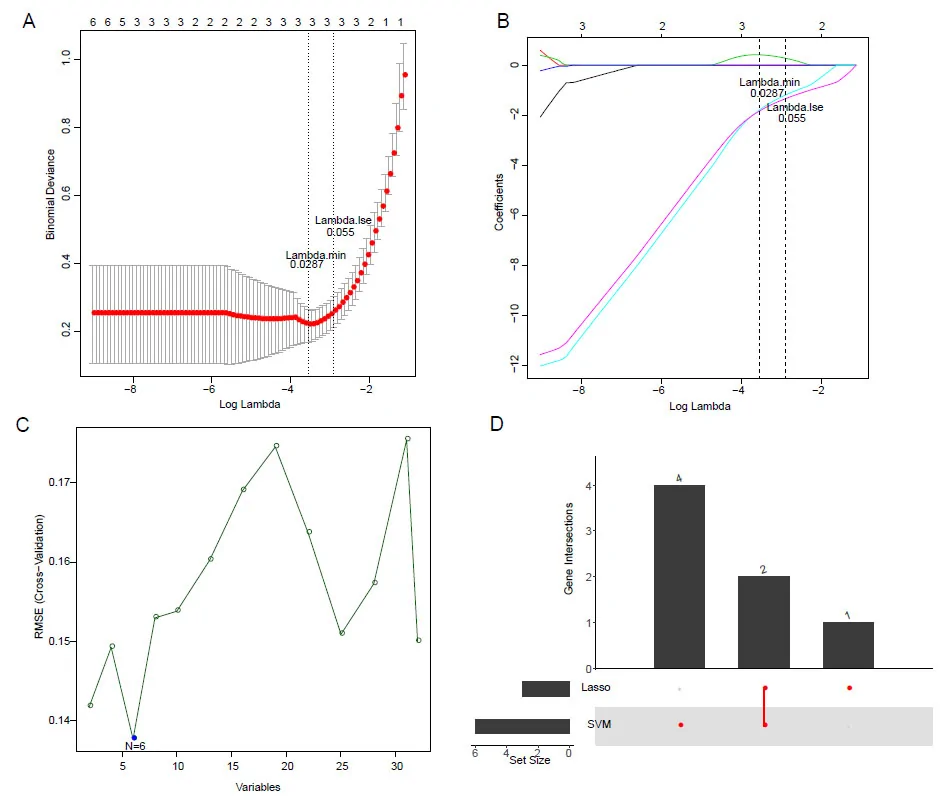
Biomarker selection by LASSO and SVM-RFE analyses. (**A**) LASSO penalty parameter plot. The abscissa represents the log(lambda) value, and the ordinate represents the degrees of freedom, which indicates the error of cross-validation. In practical analysis, the position where the cross-validation error is minimized is desired. In this plot, the position of the left dashed line indicates the location where the cross-validation error is the smallest, corresponding to lambda.min = 0.0287. Based on this position (lambda.min), the top abscissa log(lambda) is determined, and the number of feature genes, which is 3. (**B**) LASSO regression coefficient plot. The abscissa is log(lambda), and the ordinate is the coefficient of genes, demonstrating the changes in the coefficients of different variables after being penalized by λ. (**C**) Relationship plot between SVM-RFE generalization error and the number of features. The abscissa represents the number of genes included in the model for each iteration, and the ordinate represents the root mean square error. The minimum value of this number indicates the optimal model, corresponding to a gene number of 6. (**D**) Upset plot of the intersection of feature genes obtained by LASSO and SVM-RFE. There are 2 genes that are simultaneously identified as feature genes by both machine learning methods.

We performed LASSO regression analysis on the 32 candidate genes, selecting the model with the minimum error (λ.min = 0.0287), which identified three signature genes (Figs. **5A** and **B**). SVM-RFE analysis of the 32 genes revealed an optimal model with 6 signature genes at minimum error (N = 6) (Fig. **5C**). The intersection of the results from the LASSO and SVM-RFE algorithms was used to identify two biomarkers: Proline Rich 15 (PRR15) and Sushi repeat-containing protein, X-linked (*SRPX*) (Fig. **5D**).

### 3.6 TF Regulatory Network

**Fig (6):**
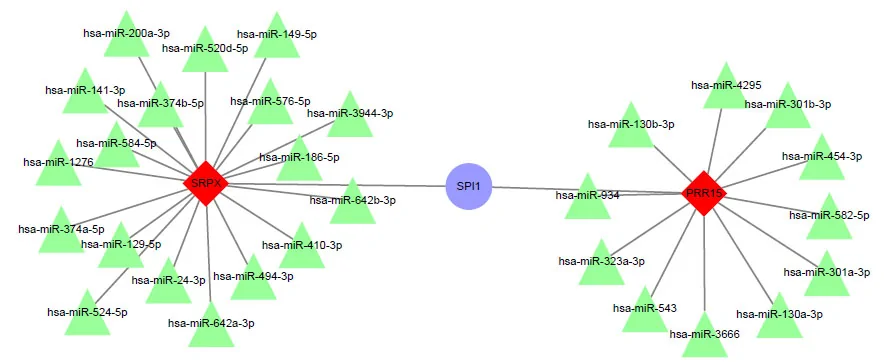
TF regulatory network. The blue circles represent TF proteins, the red diamonds represent biomarkers, and the green triangles represent miRNAs.

Prediction through the hTFtarget database showed that *PRR15* and *SRPX* shared a common TF, spleen focus-forming virus proviral integration site 1 (*SPI1*), in testicular tissue. Further investigation through the Encori database identified 11 experimentally validated miRNAs and 18 targeting *SRPX*. These miRNAs were all validated by more than one experiment. These targeted regulatory relationships were integrated to develop a targeted regulatory network, which was visualized using Cytoscape software (Fig. **6**).

### 3.7 Single-sample GSEA Analysis of *PRR15* and *SRPX*

**Fig. (7):**
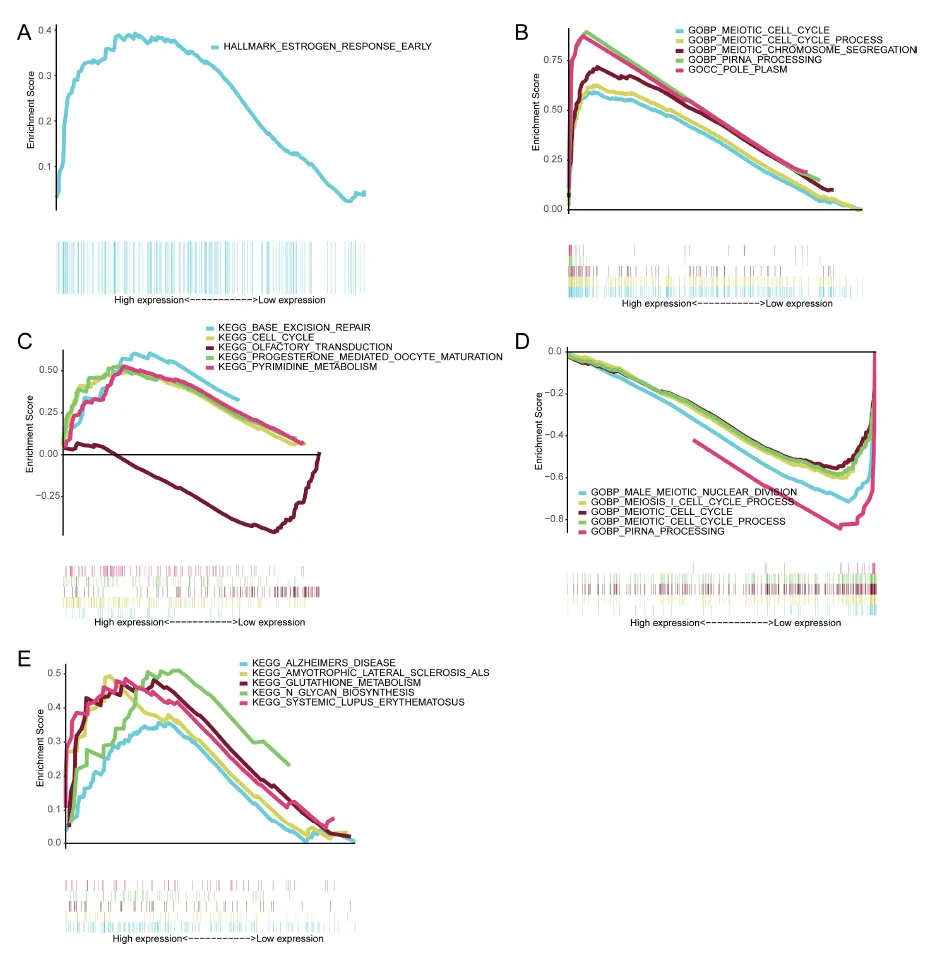
Enrichment results of GSEA analyses for *PRR15* and *SRPX* genes. (**A**) Enrichment results of the *PRR15* gene expression with respect to the early estrogen response pathway. (**B**) GO results of the GSEA analysis for the high and low expression groups of *PRR15*. (**C**) KEGG results of the GSEA analysis for the high and low expression groups of *PRR15*. (**D**) GO results of the GSEA analysis for the high and low expression groups of *SRPX*. (**E**) KEGG results of the GSEA analysis for the high and low expression groups of *SRPX*.

To identify the functional pathways associated with the two biomarkers, we stratified the samples into high-expression and low-expression groups based on their expression levels and performed GSEA enrichment analysis. It was found that the expression of the PRR15 gene was significantly associated with early estrogen response (Fig. **7A**), suggesting that the gene may be a new early estrogen response-related gene involved in the development of cryptorchidism. Additionally, the two biomarkers were primarily involved in cell division-related pathways (Fig. **7B**). *PRR15* was primarily involved in pirna processing and pole plasm, as well as the meiotic cell cycle process, including meiotic cell cycle, meiotic chromosome segregation, and was highly correlated with the progesterone-mediated oocyte maturation pathway (Figs. **7B** and **C**). The *SRPX* gene was primarily involved in male meiotic nuclear division, the meiosis I cell cycle process, the meiotic cell cycle, and piRNA processing (Figs. **7D** and **E**).

## 4 DISCUSSION

Cryptorchidism, characterized by the failure of the testis to descend normally from the retroperitoneal position in the embryo to the scrotum, is the most common congenital abnormality in male newborns [24, 25]. Cryptorchidism is one of the main causes of male infertility and may be associated with germ cell tumors [26]. Previous studies have shown that exposure to estrogen formulations can lead to various genital and reproductive abnormalities, including cryptorchidism [27, 28]. This present study investigated the role of early estrogen response in cryptorchidism. *PRR15* and *SRPX* were two cryptorchidism biomarkers closely related to early estrogen response that shared a common TF, *SPI1*. These findings expanded the therapeutic choices available to infertile men with a history of cryptorchidism. The protein encoded by *PRR15* is a low-molecular-weight protein (approximately 13.7 kDa) and highly conserved, initially discovered in the murine intestinal epithelium [29]. *PRR15* is expressed in early pregnancy human placental tissues and trophoblast cell lines, suggesting that it may play a role in the early formation of the placenta [30, 31]. Previous studies have found that *PRR15* is involved in the progression of various tumor types, including breast cancer and esophageal cancer, although the mechanism remains controversial [32-36]. The expression of *PRR15* in estrogen receptor-positive breast cancer is significantly higher than that in estrogen receptor-negative breast cancer [37], suggesting that *PRR15* may also affect the occurrence of cryptorchidism through the estrogen signaling pathway. *SRPX*, a transmembrane protein, is composed of 464 amino acids and has 3 sushi domains [38]. Relevant studies have shown that *SRPX* exhibits tumor-suppressive characteristics during cancer progression [39, 40] and also plays a role in cell apoptosis [41] and senescence [42]. It has been experimentally verified that silent *SRPX* could inhibit the migratory and invasive abilities of ovarian cancer cells [43]. In addition, increasing the dose of progesterone can upregulate *SRPX* expression in secretory endometrial tissue [44]. It can be inferred that *SRPX* may be related to estrogen response. To conclude, *PRR15* and *SRPX* were both potentially associated with estrogen response and may participate in the occurrence and development of cryptorchidism through various biological processes. However, it should be emphasized that these conclusions were based solely on computational analysis results and cannot be directly used to infer causality. Further experimental or clinical studies are needed to verify these findings.

Our TF regulatory network showed that *PRR15* and *SRPX* shared a specific TF (*SPI1*), a member of the erythroblast transformation-specific (ETS) family of TFs [45, 46]. *SPI1*, a crucial regulatory protein, plays a significant role in blood cell differentiation, the mechanism of immune cell differentiation, and tumor development [47, 48]. *SPI1* is essential for the normal development of bone marrow cells and lymphocytes. Once *SPI1* is dysregulated or knocked out, it will lead to developmental abnormalities in granulocytes, monocytes, macrophages, and B cells [49]. In recent years, numerous studies have indicated that SPI1 is associated with the progression of various tumors, such as breast cancer [50], cervical cancer [51], and endometrial cancer [52], and is also closely related to the development of endometriosis [53]. To conclude, *SPI1* influences the estrogen response and may be involved in the formation of cryptorchidism. However, the specific molecular mechanism still requires further in-depth exploration.

GSEA analysis revealed that *PRR15* and *SRPX* were mainly involved in cell division-related pathways. Estrogen, acting through estrogen receptor α, stimulates cell proliferation and tumor growth [54, 55]. Activation of the unfolded protein response (UPR) induced by estrogen is closely related to cell division. The UPR in tumor cells is activated in response to the accumulation of stress from hypoxia, therapy, and rapid cell division [56]. Moreover, the occurrence of cell division depends on the correct synthesis and folding of large amounts of protein, while the UPR can monitor and maintain the homeostasis of protein folding [57]. In addition, estrogen binds to estrogen receptors in estrogen receptor-positive breast cancer, leading to its dimerization, translocation to the nucleus, and transcriptional activity at estrogen response elements (EREs), thereby driving the cell cycle process [58, 59]. These phenomena all suggested that *PRR15* and *SRPX* may affect the estrogen response by acting on the cell division pathway. It does not have direct clinical significance, and its function and mechanism still require further verification through *in vivo *experiments.

## CONCLUSION

This study utilized public transcriptomic data and combined WGCNA, differential expression analysis, and machine learning methods to identify *PRR15* and *SRPX* as the potential molecular markers associated with early estrogen response in cryptorchidism. Our results suggest that PRR15 and SRPX may be involved in the molecular processes associated with the early estrogen response in cryptorchidism. These findings enhanced the current understanding of the molecular mechanisms underlying cryptorchidism, laying the groundwork for subsequent experimental validation. However, it should be noted that the results of this study were based on computational analysis and reflected molecular-level correlations. The findings do not have direct clinical application significance, and their functions and mechanisms still require further experimental validation.

## LIMITATIONS

This study has also certain limitations. First, the extreme scarcity of cryptorchidism tissue samples in public databases and the requirement for consistent sampling sites and sequencing platforms restricted our analysis to only two integrated datasets. This limited sample size and data source may affect the statistical power and representativeness of our findings. Future studies could enhance the robustness and generalizability of our results through multi-center collaborations to collect additional primary samples, continuous integration of newly available public data, and establishment of external validation cohorts across diverse regions and populations. Second, the absence of detailed clinical characteristics in public datasets prevented adequate adjustment for potential confounding factors. Subsequent research should prioritize obtaining comprehensive clinical information during data collection and employ multivariate or stratified analysis methods to minimize confounding effects and enhance the clinical relevance and interpretability of conclusions. Third, as our findings were obtained primarily based on computational analysis without *in vitro* or *in vivo* functional validation, causal relationships cannot be directly inferred. Follow-up studies could conduct cell and animal experiments to verify the functions of the key genes and related pathways, and combine molecular biology techniques to confirm the reliability of the results. Finally, the TF and miRNA regulatory networks predicted by this study lacked direct experimental verification and may contain biases. Therefore, in the future, methods such as ChIP-qPCR and dual-luciferase reporter assays should be performed to verify the predicted regulatory relationships and molecular interactions to further confirm the authenticity and biological significance of the regulatory networks.

## Data Availability

All data generated or analyzed during this study are included in this published article.

## LIST OF ABBREVIATIONS

TGCT: Testicular Germ Cell Tumor
HPG: Hypothalamic–Pituitary–Gonadal (HPG) Axis
INSL3: Insulin-Like Peptide 3
WGCNA: Weighted Gene Co-Expression Network Analysis
DEG: Differentially Expressed Gene
TF: Transcription Factor
GEO: Gene Expression Omnibus
Msigdb: Molecular Signatures Database
ssGSEA: Single Sample Gene Set Enrichment Analysis
KEGG: Kyoto Encyclopedia of Genes and Genomes
GO: Gene Ontology
GS: Gene Significance
MM: Module Membership
LASSO: Least Absolute Shrinkage and Selection Operator
SVM-RFE: Support Vector Machine–Recursive Feature Elimination
miRNA: MicroRNA
PRR15: Proline-Rich Protein 15
SRPX: Sushi Repeat-Containing Protein, X-Linked
SPI1: Spleen Focus-Forming Virus Proviral Integration Site 1
ETS: E26 Transformation-Specific Family
UPR: Unfolded Protein Response
ERE: Estrogen Response Element
ES: Enrichment Score
