# Supplementary material for: Analysis of the Relationship Between Early Estrogen Response and Cryptorchidism
Source: Endocr Metab Immune Disord Drug Targets. 2026 Jan 15;26:E18715303419738. doi: 10.2174/0118715303419738251111055407 (PMC13563426; doi:10.2174/0118715303419738251111055407)
Supplement: Supplementary file 1 [file EMIDDT-26-E18715303419738_SD1.pdf]

Supplementary Material

Analysis of the Relationship Between Early Estrogen Response and Cryptorchidism

Dashuai Miao[1], Yifang Sun[1], Xiang Yu[1], Suyin Feng[2,3], Long Zhu[2,3], Xiao Tong[4,*], Wenliang Ge[5,*] and Hua Li[1,*]

[1]Department of Pediatrics, Donghai County People's Hospital (Affiliated Kangda College of Nanjing Medical University), Donghai, 223000, China; [2]Jiangnan University Smart Healthcare Joint Laboratory, Donghai County People's Hospital (Affiliated Kangda College of Nanjing Medical University), Donghai, 223000, China; [3]Department of Neurosurgery, Donghai County People's Hospital (Affiliated Kangda College of Nanjing Medical University), Donghai, 223000, China; [4]Department of Pediatrics, Jiangnan University Affiliated Hospital, Wuxi, 214122, China; [5]Department of Pediatric Surgery, Affiliated Hospital of Nantong University, Nantong, China

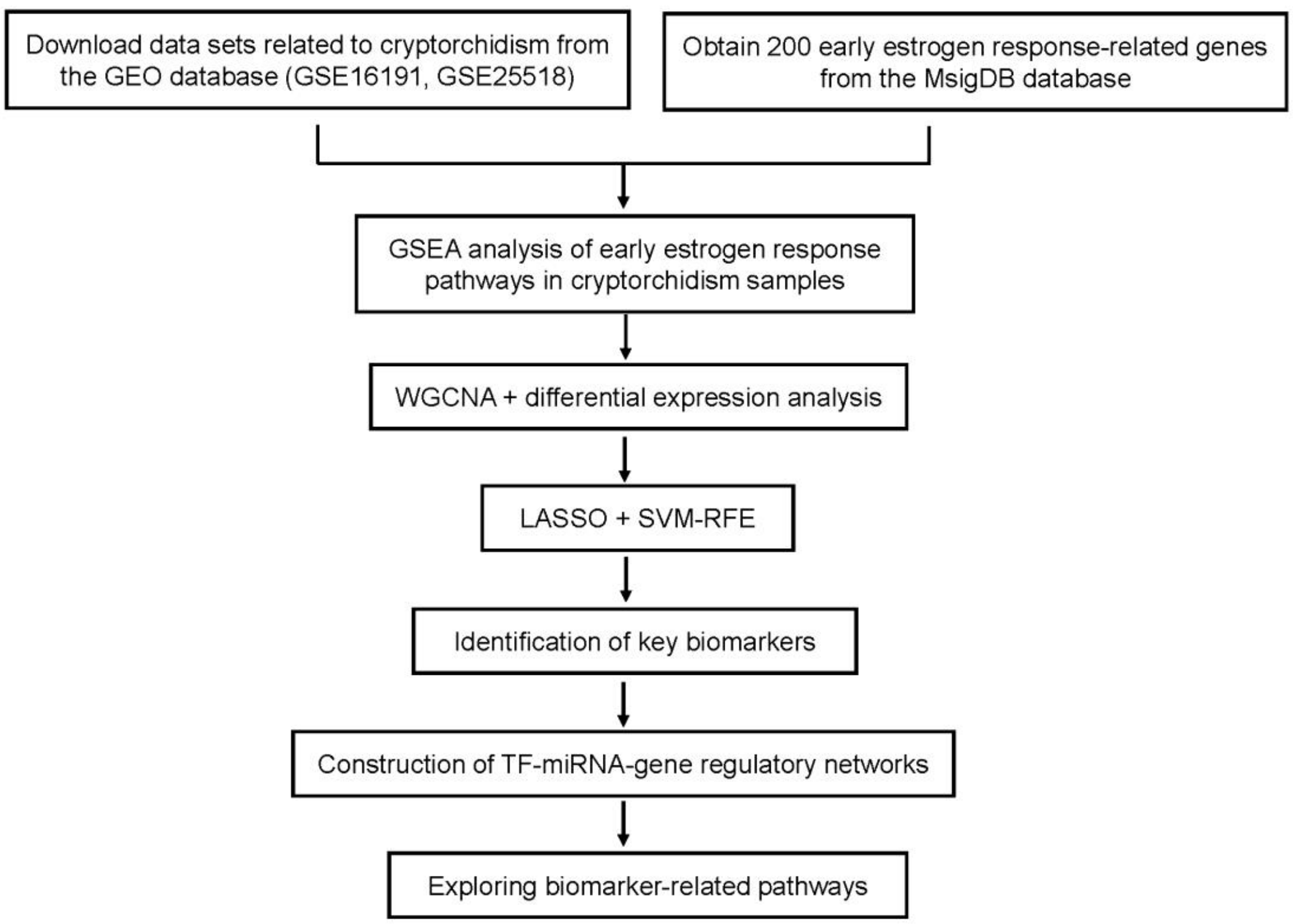

Figure (S1). Research flowchart.

Table S1. Sample information for cryptorchidism patients in the datasets used (GSE16191 and GSE25518).

**Dataset**	**Cryptorchidism**	**Control**	**Platforms**
GSE16191	16 (testicular)	4 (contralateral testicular)	GPL570
GSE25518	19 (testicular)	4 (contralateral testicular)	GPL570
